# Novel fine-scale aerial mapping approach quantifies grassland weed cover dynamics and response to management

**DOI:** 10.1371/journal.pone.0181665

**Published:** 2017-10-09

**Authors:** Carolyn M. Malmstrom, H. Scott Butterfield, Laura Planck, Christopher W. Long, Valerie T. Eviner

**Affiliations:** 1 Department of Plant Biology, Michigan State University, East Lansing, Michigan, United States of America; 2 Center for Global Change and Earth Observations, Michigan State University, East Lansing, Michigan, United States of America; 3 Graduate Program in Ecology, Evolutionary Biology, and Behavior, Michigan State University, East Lansing, Michigan, United States of America; 4 The Nature Conservancy, San Francisco, California, United States of America; 5 Department of Plant Sciences, University of California, Davis, California, United States of America; Instituto Agricultura Sostenible, SPAIN

## Abstract

Invasive weeds threaten the biodiversity and forage productivity of grasslands worldwide. However, management of these weeds is constrained by the practical difficulty of detecting small-scale infestations across large landscapes and by limits in understanding of landscape-scale invasion dynamics, including mechanisms that enable patches to expand, contract, or remain stable. While high-end hyperspectral remote sensing systems can effectively map vegetation cover, these systems are currently too costly and limited in availability for most land managers. We demonstrate application of a more accessible and cost-effective remote sensing approach, based on simple aerial imagery, for quantifying weed cover dynamics over time. In California annual grasslands, the target communities of interest include invasive weedy grasses (*Aegilops triuncialis* and *Elymus caput-medusae*) and desirable forage grass species (primarily *Avena* spp. and *Bromus* spp.). Detecting invasion of annual grasses into an annual-dominated community is particularly challenging, but we were able to consistently characterize these two communities based on their phenological differences in peak growth and senescence using maximum likelihood supervised classification of imagery acquired twice per year (in mid- and end-of season). This approach permitted us to map weed-dominated cover at a 1-m scale (correctly detecting 93% of weed patches across the landscape) and to evaluate weed cover change over time. We found that weed cover was more pervasive and persistent in management units that had no significant grazing for several years than in those that were grazed, whereas forage cover was more abundant and stable in the grazed units. This application demonstrates the power of this method for assessing fine-scale vegetation transitions across heterogeneous landscapes. It thus provides means for small-scale early detection of invasive species and for testing fundamental questions about landscape dynamics.

## Introduction

Invasive weeds substantially reduce forage production and biodiversity in grasslands worldwide [1–6], eroding their value for grazing and conservation [2, 7–10]. Despite continuous effort and resource investment [2, 11–14], control of invasive grassland weeds remains a persistent challenge in part because of the logistical demands of detecting and monitoring infestations. Critical steps in control include (1) early detection and eradication of small infestations and (2) prevention of spread to uninfested areas [15, 16]. Once an invading weed becomes established, management strategies such as prescribed burns or timed grazing are then needed to keep the weed from becoming dominant or to dissolve weed-dominant patches already formed. To be most effective, these approaches demand landscape-scale perspectives on the mechanisms underlying invasive species spread and persistence over time [17, 18]. However, most studies on the effects of management on invasive species are conducted at the much smaller scale of 1-m^2^ plots. Such small plots can assess local responses to management but provide only a limited picture of landscape heterogeneity. Moreover, unless embedded within larger landscape assessments, small plots cannot readily quantify expansion, contraction, and persistence of invaded patches over time.

Remote sensing offers means to map plant invasions at broader scales [19, 20] that complement plot-level studies. At present, mapping with remote sensing generally requires that the invaders differ from the resident community in specific ways, such as in plant chemistry (*e*.*g*., nitrogen or chlorophyll concentration), texture/morphology, phenology, or canopy level [19, 20]. Thus, while remote sensing has been extremely effective in detecting changes in functional groups of plants (*e*.*g*., woody species invading into herbaceous communities, shifts between coniferous vs. deciduous trees), it is more difficult to detect invasion of a species that is similar in functional type to the resident community it is invading (reviewed in Bradley [19]). Detecting the invasion of grasses into grassland communities is particularly challenging [21], with phenological differences so far proving to be the most helpful identifying features. However, when multiple images are required for phenological detection methods, available low- or no-cost public data has often come at a coarse spatial scale (*e*.*g*., 30-m to 1-km patch size) that fails to capture the smaller patch dynamics relevant to management [19]. The use of data from commercial satellites (e.g., 2–3 m resolution QuickBird products) in precision agriculture illustrates the power of finer-scale imagery [22], but such commercial satellite imagery has typically not been affordable for rangeland managers. Even with the limitations of coarse-scale imagery, however, some studies have been able to use differences in phenology to map invasions, based on differences in season of green-up and timing of green-up after precipitation [23], or on seasonal variation in NDVI [21].

**Fig 1:**
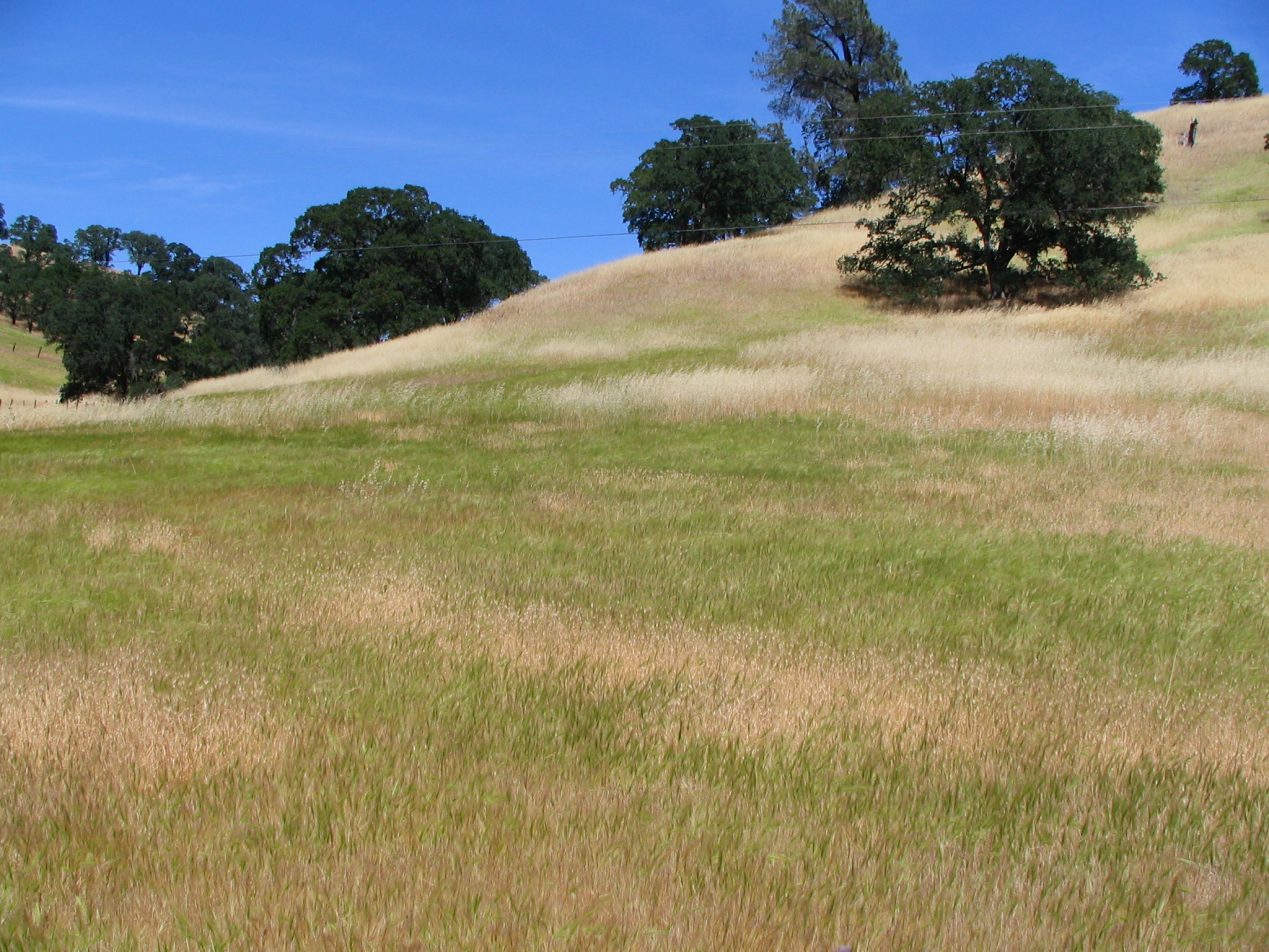
Grassland vegetation patches in may, at the beginning of the summer drought period. Light-colored senesced patches are naturalized annual forage grasses (primarily *Avena* spp. and *Bromus* spp.). Green patches are invasive weedy grasses (*Elymus caput-medusae*, medusahead; and *Aegilops triuncialis*, goatgrass).

In California’s semi-arid grasslands, two invasive weedy grasses have become particularly problematic, and are a high priority for mapping and control efforts: *Elymus caput-medusae* (L.) Nevski (medusahead) and *Aegilops triuncialis* L. (barbed goatgrass) [24–26]. These annual grasses were introduced to California in the late 19^th^ century from Eurasia [1, 27] and are now established throughout the Western United States [28]. Both species produce unpalatable forage that is avoided by livestock, particularly as the plants mature, and generate a thick, mulching layer of litter that typically persists well into the next growing season [1, 25, 29–31]. In California, broad-scale detection of these invaders is challenging because these annual grasses are invading a community already dominated by annual grass species, including *Avena* and *Bromus* spp. (wild oats and brome, valued as livestock forage). In addition, for most of the growing season, the phenology of the invasive plants overlaps with the desirable annual forage grasses. Both groups germinate with fall rains in October and November and then grow throughout the rainy winter season into the spring months [1, 29]. However, the invasive weedy grasses differ notably from the forage grasses in their end-of-season phenology. The forage grasses typically reach peak greenness in March or early April and then senesce in late April and May [31, 32], while the weedy species exhibit an extended late-season growing period that ends in late May or June [1, 33]. It is during late spring and early summer, when the invasive weedy grasses are green but the forage grasses are golden and senesced, that weed patches may be most easily identified on the ground by field observers (Fig 1).

Our study focused on two key questions: (1) How well can fine-grain phenologically-timed aerial imagery detect the invasion of medusahead and goatgrass into naturalized California annual grasslands over time? and (2) Using this method, how do the abundance and persistence of forage and weed-dominated patches vary in response to grassland management?

This study was conducted in partnership with private landowners and conservation practitioners, to assess the effectiveness of various weed mapping approaches in quantifying landscape-level impacts of land management actions (including grazing) on invasion in California grasslands [31, 32, 34, 35].

## Materials and methods

### Study system

Our study examined invasive weedy grass distribution within a 6.8-km^2^ region of semi-arid grasslands on rolling hills on the west side of the Sacramento Valley, CA, USA. The study area included four different management units on three privately-owned ranches. The landowners of these private properties gave permission to conduct this work. These units had experienced different grazing intensities over recent years, ranging from none (stocking rate of zero) to intensive rotational grazing by sheep, goats, and cattle. The two weedy grasses medusahead and goatgrass were well-established across all properties, alongside annual forage grasses (primarily *Avena barbata*, *Avena fatua*, *Bromus hordeaceus*, and *Lolium multiflorum)*. Much of the landscape matrix was thus a heterogeneous mixture of weedy and forage grasses, out of which emerged near-monospecific patches dominated by either weeds or forage. Our primary objective was to map the distribution of the strongly weed-dominated patches, which provide little forage or conservation value.

At the study location, the dominant soil types are fine smectitic thermic Andic Haploxererts; fine, mixed, active thermic Typic Palexeralfs; and fine-silty mixed, superactive thermic Typic Haploxeralfs. The climate is Mediterranean, with a cool and rainy growing season that typically begins in September and extends into May when the summer drought begins. Peak precipitation typically occurs between December and February but patterns of precipitation are quite variable. Almost no precipitation falls during summer, when mean maximum temperatures can exceed 37 ⁰C (1951–2016 data from University of California Agriculture and Natural Resources Statewide Integrated Pest Management Program, UC IPM). The two growing seasons we studied differed both in total precipitation and its temporal distribution. In growing year 2008, total annual precipitation (584 mm) was close to average (570 mm, September 1 1951 –August 31 2007), with rains heaviest in January and February and very little falling thereafter (S1 Fig). In contrast, total precipitation in growing year 2009 was only 81% of average (462 mm), with the largest rain events occurring during the shoulder seasons and little in mid-winter; small rain events occurred later into late spring and early summer than in 2008 (S1 Fig).

### Overview of approach

Our aim was to identify a robust method for mapping the distribution of weed-dominated patches that would work well even across years of different precipitation and then to use this approach to evaluate weed patch persistence or change across the four management units in our study site. The first challenge was to discern patches dominated by annual weedy grasses within the existing annual grassland, which is morphologically similar. To do this, we first characterized the phenological signature of the weeds based on subtle seasonal changes in their canopy greenness that could be discerned in contrast to the forage grasses or mixed communities of forage and weeds in which the weeds were not dominant. We considered the two weedy species (medusahead and goatgrass) as a group and did not attempt to distinguish between them. To characterize the weed group’s phenological signature, we evaluated how its greenness changed from the period of peak landscape greenness (March) to the end of the growing season (May) [34], and compared its signature to that of forage-dominated patches.

To assess vegetation greenness, we used low-cost digitized color infra-red (CIR) aerial photography familiar to many range managers (and readily replaced by low cost digital imaging technology in the future) and for which spatial resolution was fine enough (ca. 0.5 m) to resolve small weed patches in this system. From this imagery, we derived values comparable to the Normalized Difference Vegetation Index (NDVI), a classic index that identifies green vegetation [36]. We then tested different combinations of imagery and classification approaches against ground truth data to identify the most robust mapping method, and then used this method to look in detail at weed patch distribution across the study site, as detailed below.

### Image acquisition and processing

#### Acquisition and orthorectification of color infrared imagery

**Fig 2:**
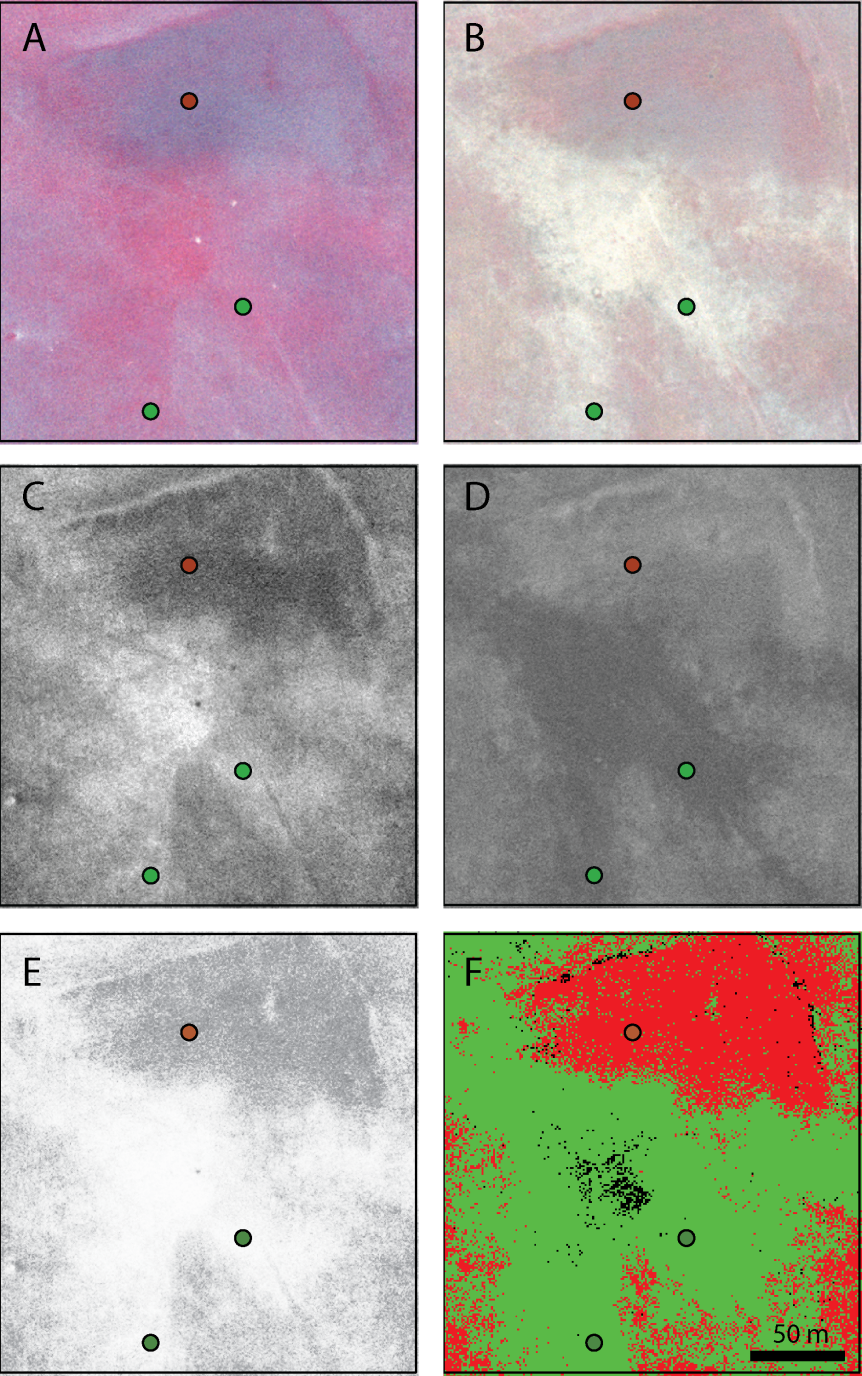
Stages in production of weed distribution maps from digitized color infrared (CIR) imagery, showing one region in detail. Close-ups of fine-scale CIR imageryin (A) March and (B) May 2008. Actively growing vegetation appears red. Normalized Difference Vegetation Index (NDVI) images derived from the CIR imagery for the same area in (C) March and (D) May. In this grey-scale NDVI imagery, actively growing green vegetation has elevated NDVI values (lighter shades). (E) the derived March–May NDVI difference image for this area for 2008;lighter shades represent greater March–May NDVI differences—areas that were greener in March than in May. (F): Maximum likelihood supervised classification based on stacked March and May NDVI. Weed-dominated vegetation is highlighted in red. In all panels, circles represent ground-truth, with red indicating weed-dominated locations and green indicating forage-dominated points.

Aerial imagery was acquired from a fixed wing airplane by Pacific Aerial Surveys (HWJ Geospatial, Oakland, CA, USA) between the hours of 12:30 and 1:30 pm, using Kodak Aerochrome III Infrared Film 1443 with a minus-blue filter at a scale of 1:34,200 or 1:35,000 (vertical shot acquired at 5,212–5,334 m flying height). The imagery was acquired with a 22.9 cm x 22.9 cm (9 inch x 9 inch) negative format from a mapping camera with a focal length of 153 mm/6 inches. Flight lines were arranged north–south with less than 5% crab and less than 2 degrees tip and tilt. For each date, we used a single image that encompassed the entire study area, so the need for image mosaicking was avoided. The images were acquired on cloud-free days at a relatively low altitude, so atmospheric corrections were also not necessary. After processing, the film was scanned without color adjustment on a photogrammetric scanner to create a digital image with approximately 0.39–0.45-meter resolution. Spring images were taken on March 10 of 2008 and 2009, during the period of peak greenness when the canopy of forage grass species is typically greener than that of the invasive weeds (Fig 2A). End-of-growing season imagery was acquired when the weed species were still green but forage grasses had senesced as judged from the ground (Fig 2B). This date varied across years due to weather-driven variation in phenology. In 2008, the May image was acquired on May 13, and in 2009, images were acquired on both May 18 (2009 A) and 26 (2009 B).

Aerial images were orthorectified in the Leica Photogrammetry Suite in ERDAS Imagine 9.3 (Hexagon Group, Sweden) using recent camera calibration reports from the United States Geological Survey (USGS) Optical Science Lab. Ground control points for orthorectification (and for ground truth work described later) were georeferenced in the field with a Trimble GeoXH (Trimble, Sunnyvale, CA, USA) using a Trimble Beacon Receiver communicating with a U.S. Coast Guard beacon and an antenna on a 2-m range pole. Point data were georeferenced in UTM10 with a WGS84 datum and then post-processed and adjusted for atmospheric conditions with reference data from the nearest National Geodetic Survey Continuously Operating Reference Station (CORS) for greater positional precision. The navigational accuracy of point locations in the field and post-processed points was approximately 0.30 m [37], which was confirmed by benchmark tests.

For effective orthorectification, a digital elevation model (DEM) is also necessary. At the time of the project, the only existing DEM for the project area was the National Elevation Dataset (NED) produced by the USGS. This DEM had a spatial resolution (pixel size) of 30 m x 30 m. This meant that an elevation value was available every 30 m across the landscape. Because the project area contained considerable landscape relief, it was determined that the NED DEM would not allow for production of accurate orthoimagery needed for concurrent analysis of several image datasets.

To ensure the accuracy of the final orthoimagery, it was necessary to create a new, more precise, DEM. The source for the elevation data was the georeferenced Digital Raster Graphic (DRG) dataset created by the USGS from the USGS7.5-minute quadrangle maps (USGS, Reston, VA, USA). Individual contours were digitized in ESRI ArcMap 9.2 as polyline features. The elevation for each contour was entered into the database table as an attribute. After digitization was complete, a 2-meter DEM was created by interpolating the elevation values of the contours. The interpolation was completed using the Topo to Raster tool in ESRI ArcGIS Toolbox 9.2. The resulting 2-meter DEM represented a large increase in elevation precision over the National Elevation Dataset and was used in image orthorectification to correct for changes in terrain.

#### Creating NDVI-type imagery

The Normalized Difference Vegetation Index (NDVI) is a simple, well-tested metric derived from red (R) and near-infrared (NIR) radiances; it is calculated as (*NIR* − *R*)/(*NIR* + *R*) [36]. Green vegetation typically shows higher values than senesced or non-living materials. To minimize topographic and seasonal differences in illumination, we created an NDVI-like image from the red and near infrared bands of each digitized color infrared image, using ENVI 4.7 (Exelis Visual Information Services Inc., Boulder, CO, USA, now a subsidiary of Harris Geospatial) (Fig 2C and 2D). The spectral properties of film differ from those of calibrated satellite instruments, but film-based NDVI estimates provide valuable utility [38]. Historically, color infrared film was designed so that the red and infrared saturation intensities appeared relatively similar to each other in order to produce an aesthetically pleasing image; as a result, NDVI values captured on film are typically lower than those captured by satellite sensors, in which the infrared saturation intensity is permitted to be greater [39]. To evaluate different methods of capturing phenological signatures, we then used Modeler in ERDAS Imagine 9.3 to produce NDVI difference images for each year as Δ*NDVI* = *NDVI*_*March*_ − *NDVI*_*May*_ (Fig 2E).

#### Mask

We created a 1-m resolution mask to remove from analysis the areas that did not include vegetation of interest (e.g., trees, water, roads, buildings, agricultural crops). We digitized masked objects in ESRI ArcMap 9.3 and exported them to ERDAS Imagine, where a 5x5 neighborhood filter was used to remove spurious data and simplify the dataset. This image was then converted to vector polygons, compared to orthoimagery, and edited as appropriate. The polygon features were then simplified in ArcMap, converted to raster, and formatted for use in ENVI.

#### Image layer stack for classification

To facilitate image classification, we created a layer stack in ENVI 4.7 using all of the NDVI and NDVI difference imagery, as well as the mask. As required for layer stacks, the imagery was resampled to a single scale (1.0 m) using cubic convolution. The increase in pixel size brought the grain of the images and that of the field evaluations of vegetation composition to a similar scale, and one that was appropriate for the degree of geopositional error in our measurements [40].

### Ground-truth data

To develop and test the classification methods examined in this study, we used ground-truth points that represented locations with known geographic coordinates in patches in which vegetation was nearly pure weeds or forage. These points were extracted according to specific criteria (detailed below) from a broader multi-year database of vegetation points that included points collected both randomly and to represent particular vegetation types or features across a large watershed. The measures at each georeferenced point included cover of key vegetation groups within 1-m radius circle, as well as individual species of interest. Cover estimates were made using Daubenmire classes: (1) < 5%, (2) 5.1–25%, (3) 25.1–50%, (4) 50.1–75%, (5) 75.1–95% and (6) 95.1–100% [41]. To create and ground truth the image classifications for our study area, we selected all data points from our vegetation database that were in our study area and for which the vegetation composition was strongly dominated either by invasive weedy or by annual grass forage species, as represented by Daubenmire cover classes of 5 or 6, or equivalent, with no other species present in substantial amounts. In effect, these “pure” points represent the purest patches of each vegetation group we could locate and thus provide the clearest characterization of vegetation properties. Such dense near-monospecific patches are also important targets of weed control efforts. We identified 98 such points for the 2008 analysis (46 weed, 52 forage), and 119 for 2009 (54 weed, 65 forage). For each year, each set of “pure” points (weed-dominated, forage-dominated) was then stratified by ranch property and half of the points within each of the three properties were randomly assigned to a single combined training data point set to support initial vegetation classification, or to a similar test set for later evaluation of classification effectiveness. This stratified approach ensured that training and test ground truth sets shared similar geographic distributions.

### NDVI characteristics at ground-truth point locations

The success of phenological-based mapping depends on the identification of phenological differences in the spectral properties of target vegetation groups. This study was motivated by field observations indicating that the invasive weedy grass species generally remain green a little longer than the forage grasses at the end of the growing season (in May) (e.g., Fig 1), although this window of difference can be very short. To characterize and differentiate the weedy grasses’ phenological signature, we compared NDVI values from the imagery at locations of known weed and forage patches in peak spring (March) and at the end of the season (May) in both study years. To test whether the signal from weed-dominated vegetation was distinct, we used repeated measures MANOVA (JMP Pro 12.2, SAS Institute Inc., Cary, NC, USA) with NDVI values in March and May as the time-repeated response variables with between-subject factors of year, vegetation type, property, and year x vegetation type and within-subject factors of month, month x year, month x vegetation type, month x property, and month x year x vegetation type. In the primary analysis, we compared values using the mid-May image for 2009 (2009 A), which was most similar to the date of image acquisition in May 2008. However, because weed senescence may occur quickly in May, we also evaluated how NDVI values differed between two dates in May 2009 (2009A: May 18, and 2009B: May 26). Finally, in addition to considering the March and May NDVI characteristics of the vegetation types, we also considered the March—May NDVI differences.

### Classification approaches

After characterizing the phenological signature of weed-dominated vegetation, the next step was to determine whether a phenological-based approach could perform consistently enough over time to serve as a reliable tool for multi-year detection of weed persistence, expansion or contraction. Our first questions centered on the choice of imagery inputs. Could a single NDVI image provide enough classification power? If so, which month for image acquisition would be best: March (mid-spring), when vegetation is most likely to be green? Or May (the end of the growing season), when weed-dominated patches are most visible to a field observer? Alternatively, would using two images improve classification accuracy enough to merit the extra costs and processing time? If so, was it most effective to stack the two images and evaluate the two-layer set (stacked NDVI) simultaneously as two bands of a single image? Or rather was it more effective to create a difference image (ΔNDVI = March NDVI–May NDVI) that would highlight the phenological changes in which we were most interested? ΔNDVI contains less information than stacked NDVI (only the difference values, not the absolute values from the two parent images are available in the stacked NDVI imagery), but that information focuses specifically on temporal NDVI changes relevant to a phenology-based analysis.

**Fig 3:**
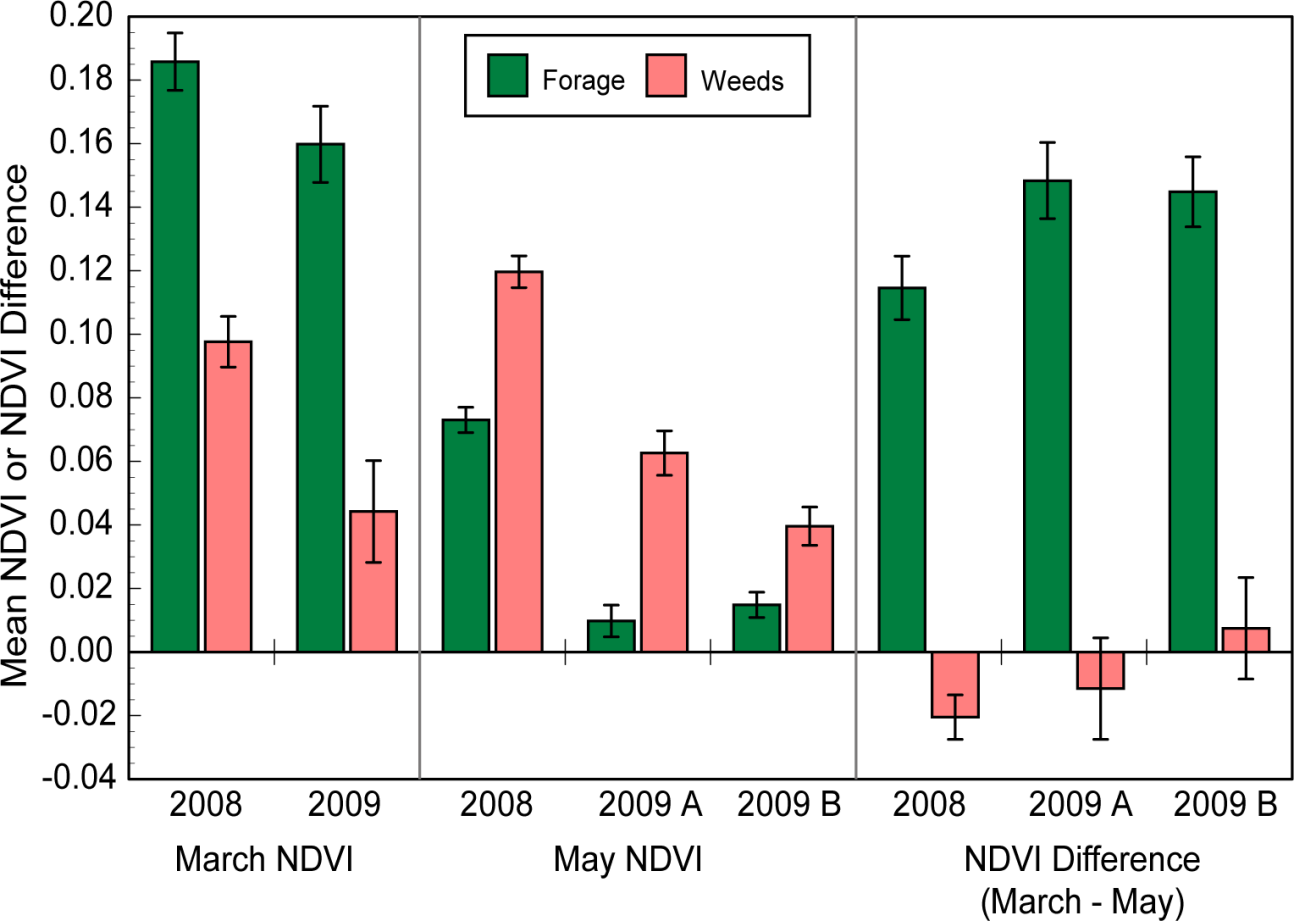
Mean NDVI values or March–May NDVI difference values extracted from imagery at locations of ground-truth points dominated by “pure” weeds or forage (>75% cover, no other significant vegetation) in the field. Error bars show standard error of the mean (SEM); n = 46–65.

To test these questions, we compared the robustness of classifications that used these four different types of imagery inputs, all used after conversion to NDVI-analogues: (1) March NDVI alone (e.g., Fig 2C); (2) May NDVI alone (e.g., Fig 2D); (3) a two-layer stack of March and May NDVI, classified together as two bands of a single image (stacked NDVI, e.g., Fig 2C and 2D); and (4) ΔNDVI_,_ a single-band difference image made, as previously described, by subtracting May NDVI from March NDVI (e.g., Fig 2E).

With these four types of inputs, we tested both unsupervised and supervised classification methods to delineate vegetation types. For simplicity, all classifications relied solely on NDVI imagery and ground truth data; none utilized additional information (*e*.*g*., slope, aspect, or soil). Throughout, the same mask was used to remove water, trees, roads, and structures from the classification. All classifications were conducted on a pixel-by-pixel basis from raster imagery with 1-m^2^ pixels.

#### Supervised classification

To conduct a supervised classification on raster data, the operator must provide the software with information necessary to determine the vegetation type represented by each pixel, often by providing georeferenced “training” sites that exemplify the properties of the target vegetation to be identified. We conducted parallelepiped supervised classifications (in ENVI 4.7) on all image sets using the training set of weed- and forage-dominated “pure” ground truth points previously described (> 75% cover of each target group and Daubenmire class values of 5 or 6). Iterative testing indicated that the most effective classifications were produced when we used a standard deviation of 2.0 for the weed classes and 1.0 for the forage classes, although a small number of pixels fell outside the standard deviation constraints and were unclassified. We conducted additional supervised classifications in ENVI using maximum likelihood (ML) classification, which required at least two image layers per analysis; the maximum likelihood classification was thus conducted only with the two-layer stacked NDVI image inputs (2008, 2009 A, 2009 B). Results were not strongly sensitive to threshold choice; we used 70% for consistency with the unsupervised approach (below).

#### Unsupervised classification

Unsupervised classification is easier than supervised classification for the operator to initiate, as the computer simply generates the specified number of map classes from imagery inputs using one of several algorithms. However, then the operator must determine which, if any, of the computer-generated map classes best represents the target vegetation type. Here we conducted unsupervised isodata classifications in ENVI 4.7 on all image sets. Each classification was run for 40 iterations with a pixel change threshold of 2.0% to create 8 classes, as previously determined in iterative tests to be effective [34]. After the unsupervised classifications were produced, we assigned vegetation types to the computer-generated map classes by comparing the distribution of the classes to the same training set of “pure” ground truth points (weed-dominated or forage-dominated) used to produce the supervised classifications. A map class was designated as weed-dominated if at least 70% of the “pure” ground truth points falling within its extent were weed-dominated points. All other classes were designated “Non-Weeds,” which included both forage-dominated pixels and heterogeneous weed-forage mixes. In a few cases, unsupervised classification produced a map class that did not contain any ground-truth points, in which case that class was assigned the identity of the majority class surrounding it.

#### Metrics for comparison of classification accuracies

To quantify classification accuracy, we compared the weed maps produced for each combination of imagery and classification approach with the “test” or “validation” set of ground-truth data points that were distinct from the training points used to produce the classification. Following the classic methods of Congalton [42], we used an error (confusion) matrix to calculate four metrics, based on the distribution of weed and non-weed class pixels and ground truth points:

(1) Overall accuracy and (2) the Kappa statistic [43] describe the general accuracy of a classification. Overall accuracy is the percentage of test points for which map classes and field data agree, across all map classes. The Kappa statistic adjusts overall accuracy to take into account agreement that might occur solely by chance. It is calculated as: (*Observed*–*Expected*)/(1 –*Expected*), where *Observed* = *Overall Accuracy*. *Expected* is calculated as the product matrix divided by the cumulative sum of the product matrix [44]. In the Kappa analysis, we used the Z-test to determine if each classification was better than random at α = 0.05 [45]. For the Kappa statistic, values above 0.60 indicate good to excellent agreement between the classification and the ground truth data [45, 46].

(3) *Producer’s accuracy* measures the percentage of a specific target vegetation on the ground that the map properly describes (i.e., how much of the target vegetation does the map “find”?). It is calculated as the percentage of ground-truth points correctly identified as the target vegetation out of the total set of ground-truth points for that vegetation type. For example, if there are 100 ground-truth points on the ground that represent weed-dominated vegetation and only 80 of them fall within the map’s weed class, then the producer’s accuracy for the weed class in that map is 80% and the map has missed 20% (omission error: 100% - 80%).

(4) *User’s accuracy* describes the purity of a specific map class. For example, if the map says that a particular area is best classified as weed-dominated, how true is this in the field? What percentage of the vegetation in the field area corresponding to the map class “Weeds” is in fact weed-dominated? User’s accuracy for a target vegetation type is calculated as the percentage of all ground-truth points within the field area corresponding to a map class that correctly match the map class type. For example, if within the field area delineated by the map’s weed-dominated class there are 80 ground-truth points representing weed-dominated vegetation, but also 40 ground-truth points representing vegetation dominated by other species or by vegetation mixes, then the user’s accuracy for weed-dominated cover is 80/(80 + 40) or 66.7%.

To identify which mapping approaches were most robust to changing environmental conditions, we calculated the accuracy metrics (overall accuracy, kappa statistic, producer’s accuracy, user’s accuracy) for each classification method (supervised, unsupervised) x NDVI image input type (March only, May only, stacked March + May, March–May difference) combination for each of the two years (2008 and 2009) and the two May dates (2009A, 2009B). For each combination, we then calculated the mean and coefficient of variation (CV) of the accuracy metrics that resulted from using imagery inputs from these different dates. In our judgement, the best mapping approach would combine high accuracy (i.e., high mean kappa statistic) with strong consistency (i.e., low CV of the kappa statistic), which would support its application in study of cover changes over time.

### Evaluation of grazing influence on weed cover dynamics

After determining which mapping approach had the greatest and most consistent accuracy, we used this approach to compare weed distributions in 2008 and 2009 across the study site. We evaluated the percentages of the landscape that were dominated by weeds in each year and how much gain or loss of weed-dominated area occurred between years. We then analyzed cover distributions by management unit. The study site included four separate management units that represented a serendipitous pre-existing gradient of grazing intensity from west to east. At the time of imagery acquisition, the westernmost management unit (M1) was used for turkey hunting and for more than five years had experienced no grazing, except by an occasional animal that broke through a neighboring fence. A second central management unit (M2) on a separate property had likewise been set aside for most of the preceding five years, and had only been grazed briefly on a few occasions by a small number of sheep. In contrast, another unit (M3) on that same property had been moderately grazed by sheep and cattle on a regular basis; and the eastern unit (M4) had been moderately to intensely grazed for more than 8 years by sheep, cattle, and goats, largely with managed intensive rotational grazing. For M3 and M4, the estimated mean stocking rates were 0.4–1.3 animal units (AU) ha^-1^; short-term stocking rates in sub-areas of M4 were higher during rotations.

As a case study to evaluate application, we used the most effective mapping approach to compare the extent of weed-dominated cover in the largely ungrazed management units (M1 and M2) with that in the regularly grazed units (M3 and M4). While the units we studied were not established with experimental research in mind and replication was limited, each grazing category (largely ungrazed, regularly grazed) spanned similar soils and topography and included management units from two different properties. Moreover, the study site offered an opportunity for realistic application: the management units were large and part of independent working ranches managed for diverse commercial purposes.

## Results

### Vegetation phenology

Individual digitized CIR images provided clues about weed patch distribution that could be used by land managers in preliminary assessments to identify suspect areas to investigate on the ground. In March CIR images, for example, vegetation patches associated with forage-dominated ground truth points (Fig 2A, green circles) generally appeared greener (traditionally visualized as red in CIR imagery) than those associated with weed-dominated points (Fig 2A, red circle). In May CIR imagery, the opposite was true. While less greenness was evident overall, because the landscape was drying, the vegetation patches associated with weed-dominated ground truth were greener than forage-associated patches (Fig 2B).

However, for stronger identification power, our results demonstrate the value of standardizing imagery to a single spatial scale (it can be difficult to obtain multiple aerial images at exactly the same spatial scale) and to NDVI units, which help correct for differences in illumination. In the northern hemisphere, for example, solar zenith angles are greater in March than in May, and thus produce more notable hill-shadows, which can make interpretation more difficult (cf. Fig 2A vs Fig 2B). These hill shadow effects were essentially eliminated with NDVI (Fig 2C, Fig 2D)—because it is calculated as a ratio of radiation types—resulting in cleaner comparisons of imagery time series and stronger identification of the phenological signatures of vegetation over time. This allowed us to use two images per year with more confidence, either as stacked NDVI or difference NDVI (Fig 2E), from which final classifications produced easy-to-read maps (Fig 2F).

We analyzed the standardized NDVI imagery to quantify the phenological signatures of our target vegetation types. We extracted NDVI values from imagery at known locations dominated by “pure” forage or weeds (>75% cover, no other significant species). In comparisons of mid-March and mid-May values, we found significant month x vegetation type effects as predicted (*F*_1,211_ = 155.1, *P* < 0.0001), indicating that the annual weed grasses could be distinguished from the annual forage species by the nature of the change in their spectral properties over the growing season. In March (mid-growing season), mean NDVI values in weed-dominated patches were less than half those in forage patches, whereas in May (end of the growing season) the opposite relationship was evident: weed patch values were about 2-fold greater than forage values (Fig 3). However, the highest NDVI values at weed-dominated points (seen in May) only reached 54% of the highest values at forage-dominated points (seen in March). The NDVI difference imagery (March–May) provides one simple way to capture these distinct temporal signatures of the vegetation types and showed greater difference between them than either March or May NDVI values alone (input type x vegetation type effect: *F*_2,210_ = 90.6, *P* < 0.0001).

The NDVI values at ground-truth points showed additional features consistent with the system’s field ecology, which increased our confidence in the biological value of the digitized imagery. Three examples illustrate this point. First, in the 2009 drought year, NDVI values were reduced at both weed-dominated and forage-dominated ground truth points (*F*_1,211_ = 44.5, *P* < 0.0001), with the biggest reductions in NDVI evident in May (month x year effect, *F*_1,211_ = 5.88, *P* = 0.0162) (Fig 3), which was an effect consistent with our general field observations. Second, we also found that NDVI values at ground truth points varied by property (property effect: *F*_2,211_ = 37.2, *P* < 0.0001), in a manner consistent with our field impressions. The lowest mean NDVI values were seen on the least-grazed ranch, particularly in March (month x property effect: *F*_2,211_ = 7.11, *P* = 0.001; least square means of 0.078 vs 0.143 and 0.157). This property had extensive patches of weedy grasses with thatch accumulation, so the low NDVI values are consistent with the biological likelihood that thatch build-up obscured the detection of the green biomass in spring, or actually suppressed it, as seen in Bartolome, Stroud [47]. Finally, comparison of the two different dates of image acquisition in May 2009 (2009A: May 18, 2009B: May 26) captured the field-observed quick decline in greenness of the weed-dominated cover in that short period (May date x vegetation type interaction, *F*_2,113_ = 7.01, *P* = 0.0092) (Fig 3), illustrating the ephemeral nature of that signal.

### Classification accuracy

The landscape maps of weed patch distribution that we produced differed somewhat by method and input. To assess classification effectiveness, we considered four different accuracy metrics. In general, we found that approaches that used inputs (ΔNDVI and stacked NDVI) derived from imagery collected at two different times per year (i.e., March and May) were more accurate and consistent across years than those that used image inputs from one month alone.

#### Overall mapping accuracy & kappa values

**Fig 4:**
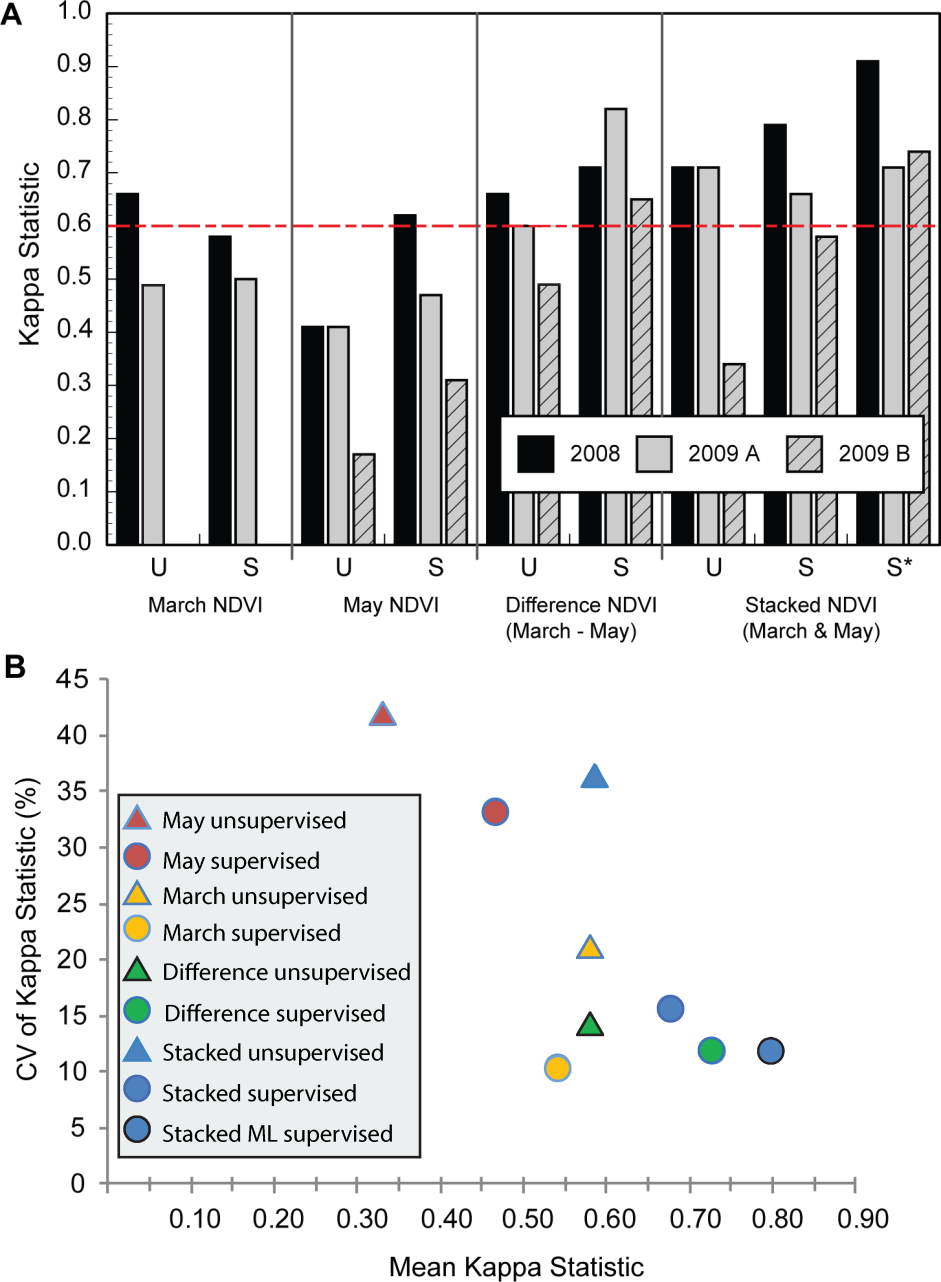
Comparison of kappa values of classifications for four types of NDVI-analogue image inputs processed with both unsupervised (U) and supervised (S) classification algorithms. ‘S’ classifications were conducted with a parallelpiped approach; ‘S*’ was conducted with maximum likelihood methods. Values > 0.60 (dashed red line) are considered good to excellent. (B) Mean and coefficient of variation (CV) of Kappa statistic for each combination of classification method and image input. In legend, “Stacked” indicates stacked March and May NDVI imagery; “Difference” indicates March–May NDVI difference image; “ML” = maximum likelihood.

*Overall accuracy* describes the percentage of all ground-truthed patch types that were correctly identified across the entire mapping area. The *kappa statistic* is a more rigorous metric, which adjusts overall accuracy for the probability that some of the patches could be correctly identified based on pure chance. Analysis of the distribution of overall accuracy and kappa statistic values for the different mapping approaches indicated that the most important determinant of classification success was the choice of input (Fig 4A, S2 Fig). For example, we used least squares ANOVA to model the kappa values from all classifications (except those derived from May 2009 B data, which were excluded to balance data across years) as a function of the fixed effects of year (2008, 2009), classification method (unsupervised, parallelpiped supervised, maximum likelihood supervised), and image input type (March, May, Difference, or Stacked NDVI) (model *R*^2^ = 0.822, *F*_6,11_ = 8.44, *P* = 0.0013). In this model, image input type had a notably greater effect on kappa statistics (*F*_3,11_ = 9.56, *P* = 0.0021) than did either year (*F*_1,11_ = 4.53, *P* = 0.0568) or classification method (*F*_2,11_ = 2.82, *P* = 0.1028).

Contrast tests among image input types found that classifications derived from time-series inputs (i.e., ΔNDVI or stacked NDVI inputs) containing information from both March and May notably out-performed those based on single images per year (contrast, *F*_1,11_ = 26.2, *P* = 0.0003). Kappa values for classifications based on time-series inputs exceeded 0.6, which describes good to excellent classifications (least squares means: stacked NDVI 0.753 and ΔNDVI 0.733) whereas those based on single images did not (least squares means: March alone 0.593 and May alone 0.513) (Fig 4A). Results for overall accuracy were similar (S2 Fig), with classifications using time-series imagery more readily achieving 80% accuracy (excellent).

As noted, year effects in this model explained an additional but smaller portion of variability in kappa values. Mean kappa values were marginally greater during 2008 when precipitation was average than in the 2009 drought year (least squares means: 2008, 0.686; 2009, 0.611; *F*_1,11_ = 4.53, *P* = 0.057). Although classification method itself did not significantly explain additional variability in kappa values (*F*_2,11_ = 2.81, *P* = 0.103), mean kappa values were highest for maximum likelihood supervised classification (least squares means, 0.720) and lowest for unsupervised classification (least squares mean, 0.581).

In a second similar analysis, we considered how kappa values for 2009 classifications were influenced by the choice of May image acquisition date, using a modified model with fixed effects of May date (A, B), classification method, and image input type (model *R*^2^ = 0.888, *F*_5,8_ = 12.65, *P* = 0.0012; classifications based on March data alone were omitted). In this model, the effects of classification method and image input type were both significant (*F*_2,8_ = 6.66, *P* = 0.0198 and *F*_2,8_ = 14.8, *P* = 0.0020, respectively). More notably, classifications based on the later May date had significantly poorer kappa values (the least square mean for May A was 0.653 and 0.496 for May B; *F*_1,8_ = 12.91, *P* = 0.007) (Figs 4A and S2). Moreover, the lowest kappa value (0.17) and the only statistically unsupported classification (*Z*-value = 0.93) were based on May B imagery.

In our analyses, we sought to identify a mapping approach that would both achieve high mean kappa and perform consistently well across time. Thus, for each approach we also evaluated the coefficient of variation (CV) across time as a function of mean kappa, and judged the best performers to be those with both high mean kappa values and low kappa CV (Fig 4B). This evaluation underscores the value of classification based on multiple seasonal NDVI images (stacked NDVI or ΔNDVI), as opposed to one-time shots. Contrary to initial field-based intuition, classifications based solely on May images were the worst performers (lower kappa, higher CV).

**Fig 5:**
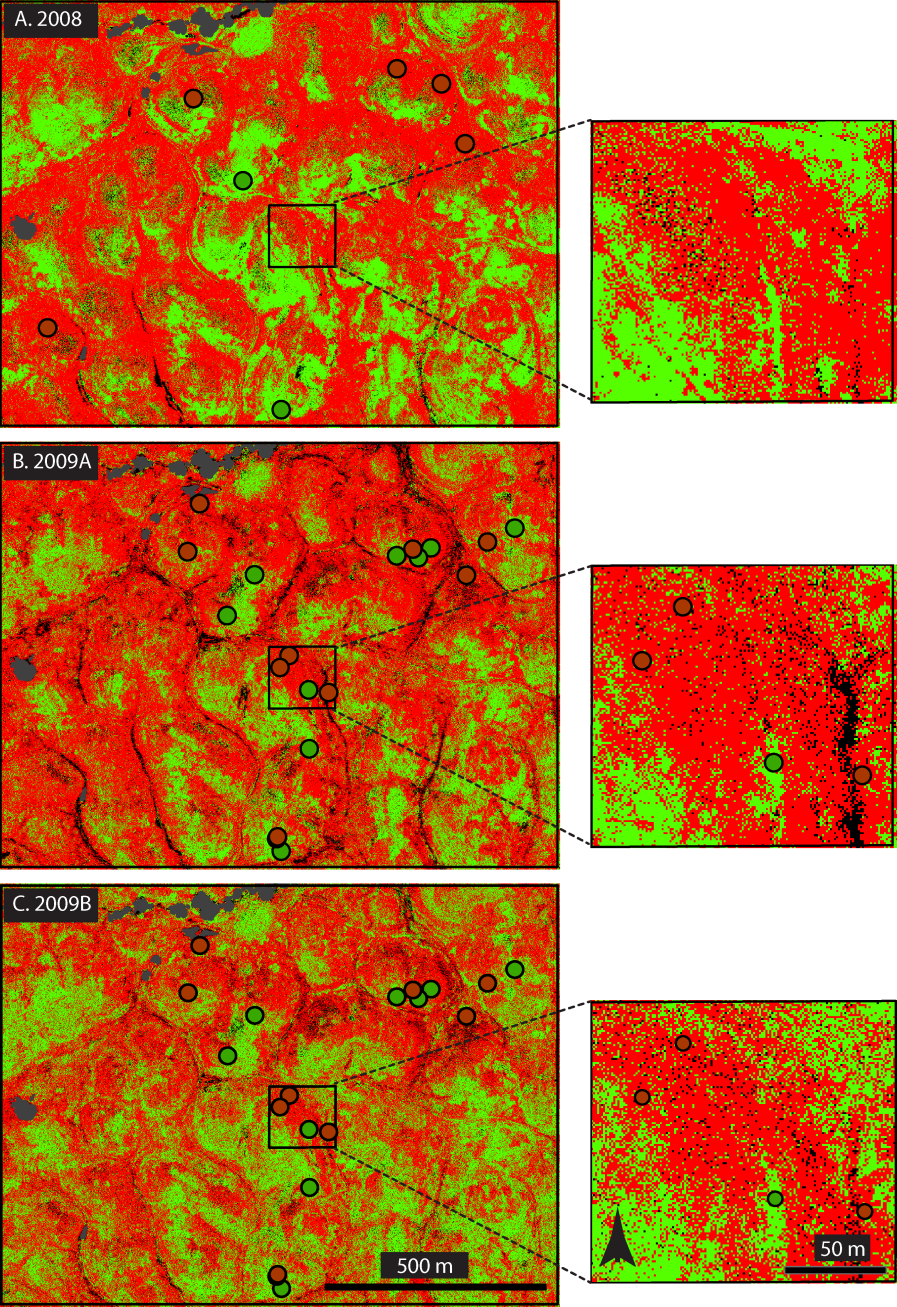
Maximum likelihood supervised classification based on stacked March and May NDVI-analogues. Each large panel shows the same area as mapped based on different imagery (A) 2008, (B) 2009 A, and (C) 2009 B. Red represents weed-dominated cover, green represents forage-dominated cover. Circles with corresponding color represent ground truth from the same year. Fine detail is magnified in excerpts to the right of each panel.

By our criteria, the best performer was maximum likelihood supervised classification of stacked NDVI (kappa = 0.73–0.92) (Fig 4B). Maps produced by this approach showed fine details of weed- and forage-dominated areas and corresponded well to ground truth (Figs 2F and 5A–5C). Even the “runner-up” methods produced valuable classifications, particularly when based on two images. The degree of detail captured by these maps was notable. In additional informal tests, we loaded maps onto our GPS units and were able to walk through the grasslands while watching in real-time our progress across the digital maps. The consistency of the maps and the actual landscape was markedly high.

One of the features that these maps captured was the persistence (or stability) of large nearly monospecific weed-dominated patches in the ungrazed management units, which we also observed in the field but could not quantify over a large area without the assistance of remote sensing methods. As illustrated (Fig 5), some of these patches were tens, sometimes almost hundreds, of meters across, and varied little across the two years, likely because of the persistence of accumulated thatch and thatch feedbacks (Fig 5A and 5B) [48]. In this ungrazed, weed-dominated landscape, distinct forage patches were also evident (Fig 5A) but during the drier year (2009) these patches lost some integrity and were invaded by the weeds (Fig 5B). Comparison of May imagery dates (Fig 5B and 5C) shows that choice of date might influence how the borders and homogeneity of patches were perceived, but general patch patterns remained recognizably similar.

#### Weed mapping accuracy

**Fig 6:**
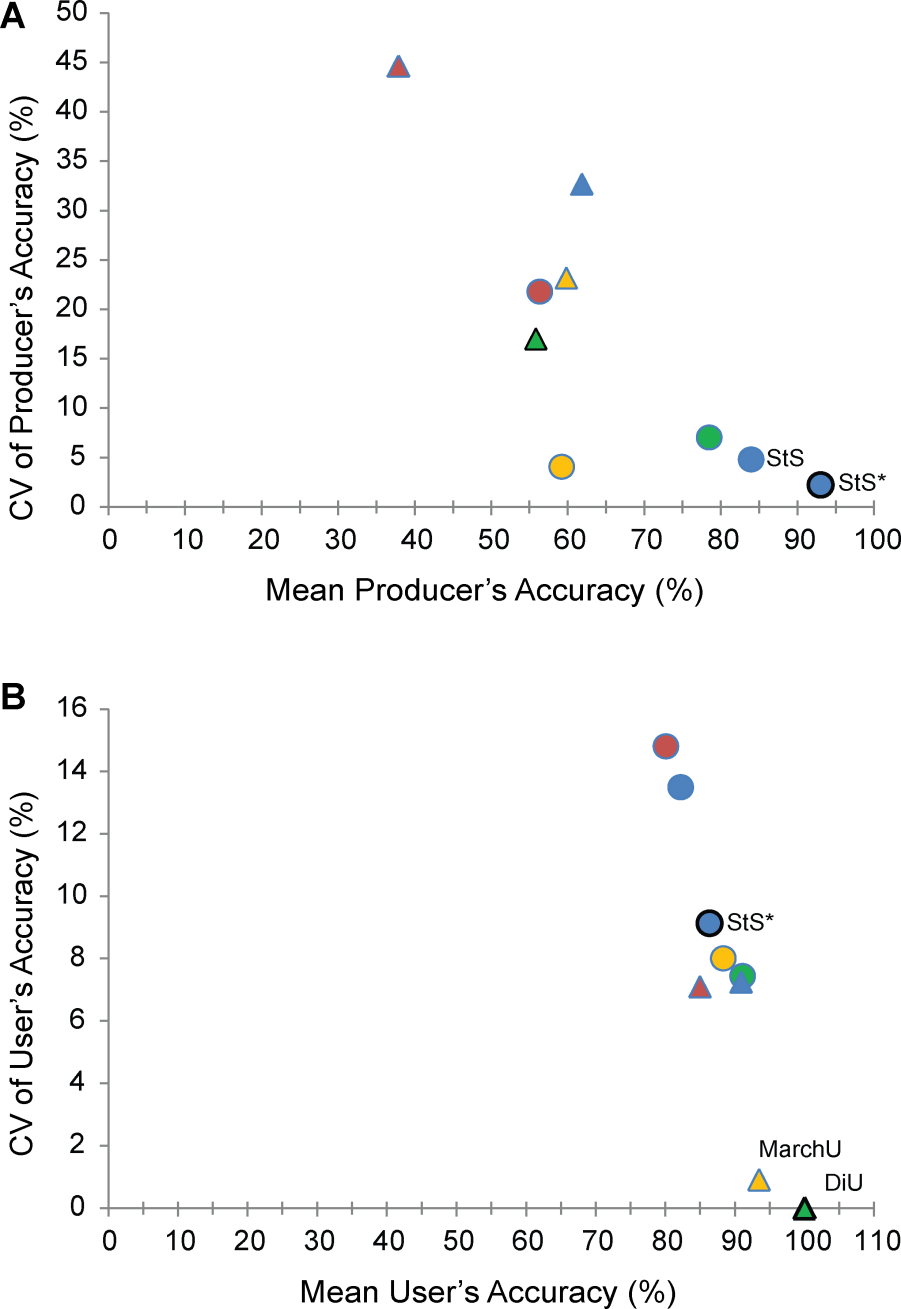
**Means and coefficients of variation for (A) producer’s and (B) user’s accuracy for identification of weed-dominated vegetation, for each combination of classification approach and image inputs.** Image inputs: warm colors, single image inputs (red = May, yellow = March); cool colors, double image inputs (blue = stacked March & May, green = March–May difference). Classification types: triangles, unsupervised; circles, supervised. StS* indicates ML supervised classification of stacked NDVI imagery. StS indicates parallelpiped supervised classification of the same. MarchU indicates unsupervised classification of March imagery. DiU indicates unsupervised classification of NDVI difference imagery.

Since our primary aim was to map weed cover, it was vital to assess the probability of locating all of the existing weed-dominated vegetation known from ground-truth when using a given map (producer’s accuracy). The percentage of weed-dominated cover a map misses can then be estimated by subtracting the producer’s accuracy from 100%. As with the kappa statistic, we considered both the mean and the CV of the producer’s accuracies for the different mapping approaches over time. In this evaluation, the maximum likelihood supervised classification conducted on stacked NDVI was again the strongest performer, with mean producer’s accuracy of 93% and CV of 2.2% (Fig 6A, S3 Fig), indicating this approach was likely to miss only 7% of weed-dominated cover. Parallelpiped supervised classifications of stacked NDVI and ΔNDVI were consistent performers as well.

The capacity to map all existing weed-dominated cover (producer’s accuracy) must be balanced with the chance of misidentifying other vegetation as weed-dominated, so we also considered the user’s accuracy for weed-dominated cover. User’s accuracy estimates the purity of on-the-ground vegetation within the mapped class, and the percentage of other vegetation misidentified as weed-dominated can be determined by subtracting the user’s statistic from 100%. Mean user’s accuracy for all classifications was good (> 80%) with low CV (< 15%) indicating that the maps generally did not misidentify other vegetation as weed-dominated (Fig 6B, S4 Fig). For the maximum likelihood supervised classification—the best performer in terms of kappa and producer’s accuracy—the mean user’s accuracy was strong (86.1%) as well. The strongest performer for this metric was unsupervised classification of difference imagery, with a user’s accuracy of 100%.

### Weed cover dynamics over time and influence of grazing

**Fig 7:**
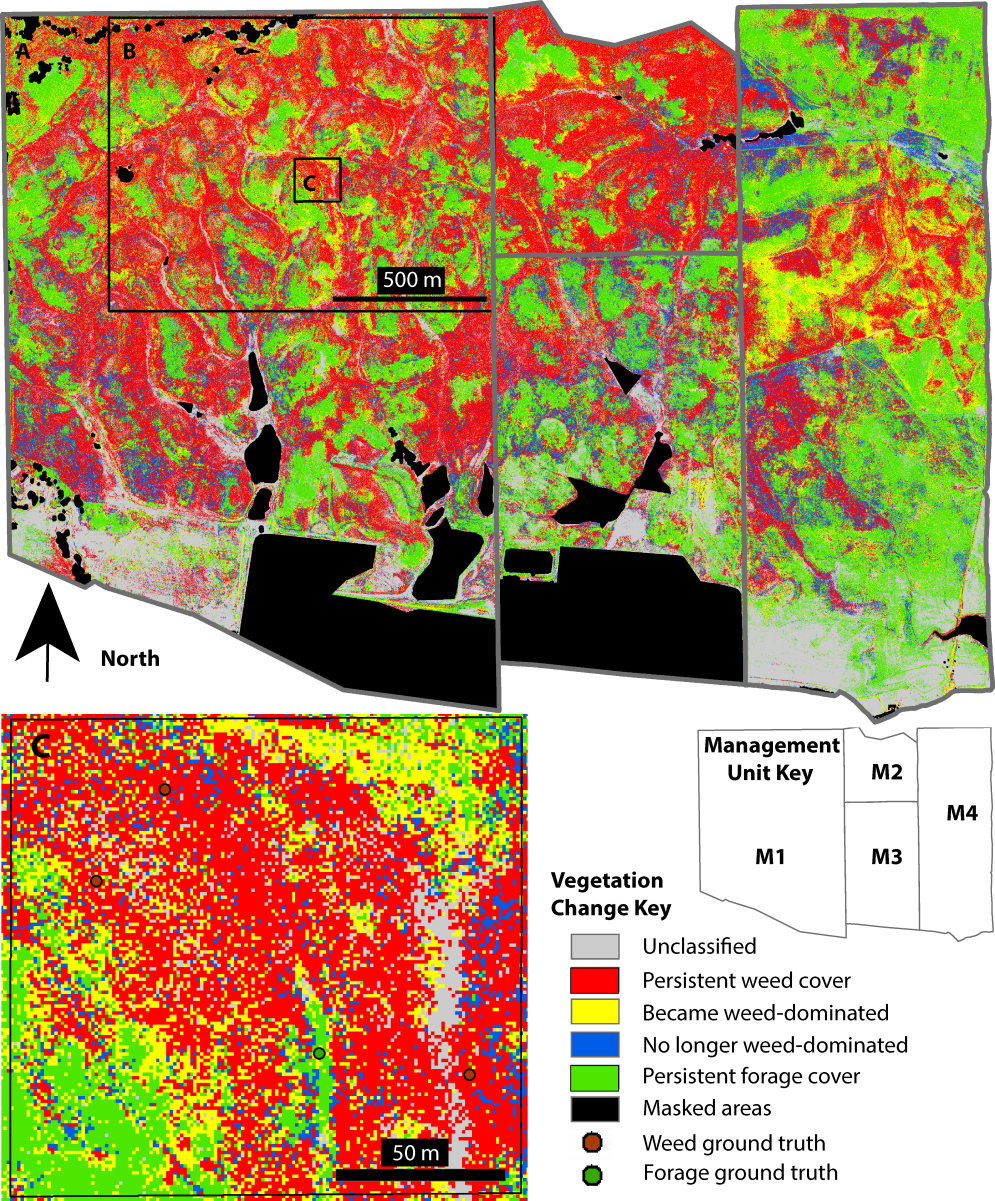
Change in weed-dominated cover from 2008 to 2009 as estimated from maximum likelihood supervised classifications of stacked NDVI inputs. (A) The full study area covering four management units (M1–M4, see key) on three independent properties. (B) The same area shown in Fig 5. Inset box C is shown enlarged below. Red and green colors indicate areas persistently dominated by weeds and forage, respectively. Yellow indicates areas of increased weed dominance; blue indicates areas of weed reduction.

Once we had identified the most accurate and consistent mapping approach (maximum likelihood supervised classification based on stacked NDVI inputs), we applied it to our study site landscape, which included four management units from three different independent ranches. We evaluated the distribution of weed-dominated cover in both 2008 and 2009, and the nature of any changes between years. In this analysis, 11.6% of the 6.8-km^2^ study landscape contained non-target land cover types such as trees, row crops, or non-vegetated surfaces (ponds, roads, etc.), which we masked in the imagery and did not examine. The remaining landscape area represented grasslands, which was categorized as weed-dominated, forage-dominated, or neither (unclassified). We then combined the vegetation maps from 2008 and 2009 to evaluate the nature of vegetation change or stability (Fig 7). The geopositional correspondence of the annual maps was excellent, due to the effective positional accuracy of the ground control points (ca. 0.30 m), the fine-grain (ca. 0.5 m) nature of the original imagery, use of a fine-scale (2-m) digital elevation model in image orthorectification, and uniform upscaling to 1-m pixels for NDVI image production.

**Fig 8:**
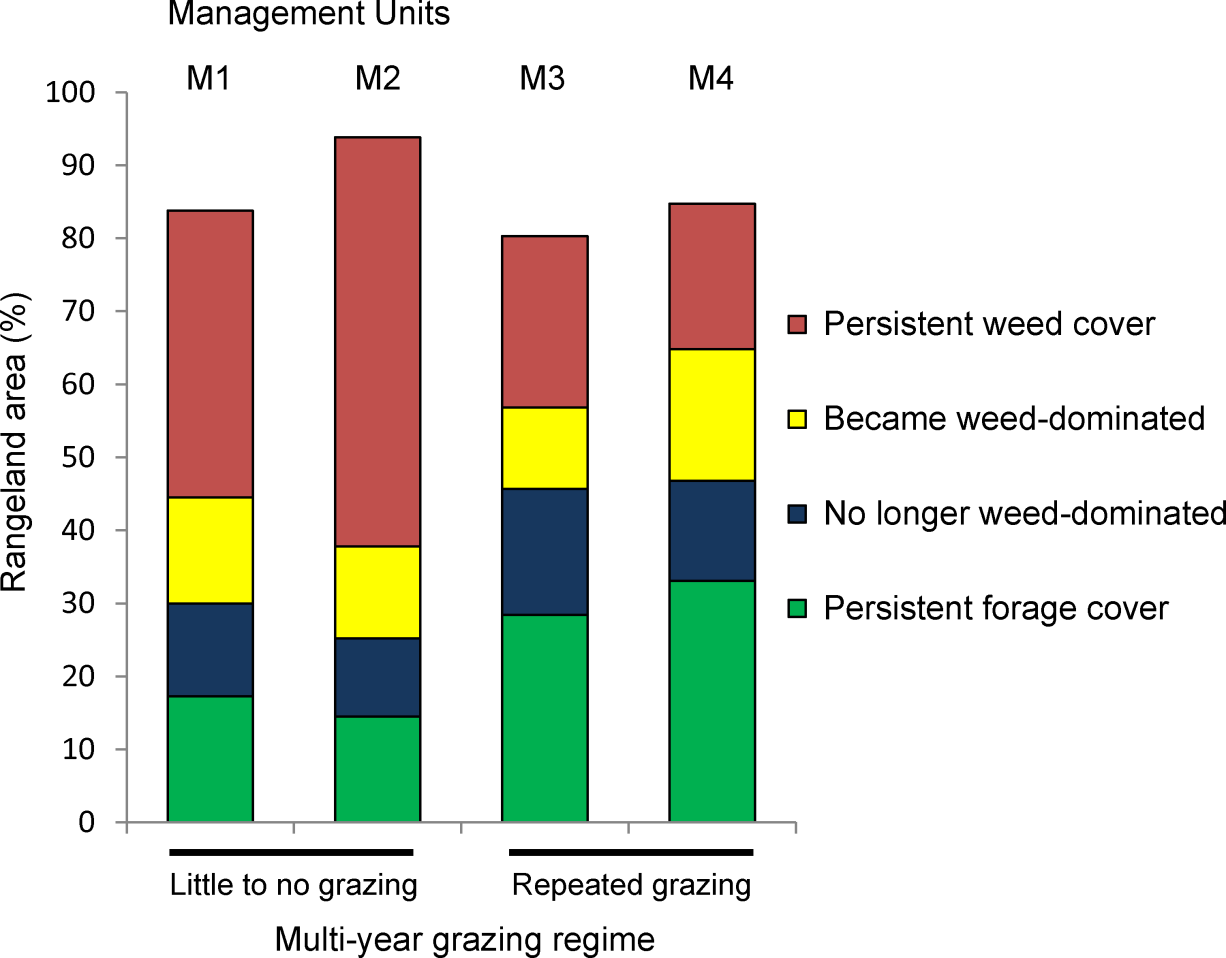
2008–2009 vegetation change patterns (% non-masked area) by management unit and grazing regime. Red and green colors represent stable areas of weed- or forage-dominated cover respectively. Yellow represents area that become weed-dominated; blue represents area of weed reduction.

Together, the fine-scale individual year maps and vegetation change map provide opportunities for numerous rich analyses. Overall, assessment of 2008–2009 vegetation change showed that 56% of the grassland (unmasked) area across the study site remained dominated by the same vegetation type (either weeds or forage) in both years, while 28.3% converted from one type to another, and 15.4% was unclassified in at least one year (Fig 7). To demonstrate applications, we highlight some aspects of how weed cover dynamics varied among management units and in response to interannual environmental variability. Most notably, the vegetation change map (Fig 7) and summary values (Fig 8) indicate that the proportion of weed-dominated cover in both 2008 and 2009 varied significantly among management units (2008: Df = 3, Likelihood Ratio, χ^2^ = 267899, *P* < 0.0001; 2009: Df = 3, Likelihood Ratio, χ^2^ = 272518, *P* < 0.0001). In Figs 7 and 8, cover that was weed-dominated in 2008 is the sum of the red + blue areas; cover that was weed-dominated in 2009 is the sum of the red + yellow areas. Specifically, in both years weed cover dominated proportionally more of the grasslands in management units with no significant grazing (M1: 52–54%; M2: 67–69%, for 2008 and 2009 respectively) than in units with repeated grazing (M3: 41–35%; M4, 33–38%) (analysis of means for proportions, ANOM, α = 0.001. M1 & M2: exceeded upper limit; M3 & M4: exceeded lower limit, for both years [49]). When grazing was absent, large near-monotypic weed patches dominated, often with organic shapes that followed landscape contours. In the field, these weed patches were associated with accumulated layers of thatch. The influence of management on weed accumulation is highlighted by fence-line contrasts, such as the border between M3 and M4 (Fig 7), for which soil types are identical on each side. Close analysis of patch details (Fig 7B) found that many weed patches spread outwards in bands at their margins during the 2009 drought year (yellow pixels), while smaller and more scattered forage patches (blue pixels) sometimes emerged within them. In contrast, weed patches in grazed units were more varied in shape and location. In the grazed units, areas of weed patch emergence were concentrated in some regions, suggesting that emergence was further influenced by within-unit management (Fig 7B). For example, vegetation patterns within M4 are influenced by use of electric fences within the unit to demarcate rotational grazing sub-units (e.g., see the diagonal line contrast starting on the unit’s western fence-line, south of the border between M2 and M3).

Without grazing, weed-dominated cover was not only more pervasive but also more persistent. In ungrazed units, for example, 76–84% of total 2008 weed cover persisted into the next year, whereas in grazed units only 58–59% did (ANOM, α = 0.001. M1 & M2: exceeded upper limit; M3 & M4: exceeded lower limit). In parallel, the percentage of persistent weed-dominated area (non-masked) was 1.6–2.8 times greater without grazing (39–56%) than with it (20–24%) (ANOM, α = 0.001. M1 & M2: exceeded upper limit; M3 & M4: exceeded lower limit). The opposite was true with forage-dominated cover: 65–72% of total 2008 forage cover persisted into the next year with grazing but only 53–54% without (ANOM, α = 0.001. M1 & M2: exceeded lower limit; M3 & M4: exceeded upper limit). Similarly, persistent forage-dominated cover was 1.6–2.4 times greater with grazing (28–33%) than without (14–17%) (ANOM, α = 0.001. M1 & M2: exceeded lower limit; M3 & M4: exceeded upper limit). As a result of these differences, the ratio of stable forage- to stable weed-dominated cover ranged from 1.2–1.6 in grazed units but only 0.25–0.44 in ungrazed units (Fig 8).

## Discussion

A key goal in ecological research is to understand what factors control the spatial distribution of vegetation patches and whether patches persist, expand, or contract over time [50–52]. Despite technological development, data to assess fine-grained patterns of vegetation turnover at the landscape scale remain limited. This information gap hampers efforts to establish and prioritize management options for invasive species control [17, 18]. Landscape-scale information about patch dynamics within grassland communities is particularly lacking, because many grassland shifts occur between species of the same functional type and have been difficult to detect remotely (reviewed in Bradley [19]).

California’s grasslands have been dominated by naturalized exotic forage grasses for 250–300 years, but nonetheless serve as quintessential examples of “working landscapes” [53] that both support rich biodiversity and provide essential rangeland feed for California’s livestock industry [54]. However, the more recent invasion of two noxious weedy grasses—*Elymus caput-medusae* and *Aegilops triuncialis*—into the naturalized forage grass-dominated community has reduced biodiversity [55], and harmed forage production and livestock welfare [1]. Thus, both weed species are targets for control by for conservation professionals and ranchers alike. Critical information needed to support adaptive management includes information about how to optimize livestock grazing for weed control and how to assess the impacts of treatments over large areas and longer time [26].

Remote sensing has offered promise as a tool for grassland assessments, but it has proven particularly challenging to distinguish among groups of similar grasses [21] and some methods—such as use of hyperspectral imagery (reviewed in [56])—are currently beyond the reach of most managers. To meet the pressing need for information to evaluate management strategies, we thus developed and tested a relatively simple phenologically-based remote sensing approach that used aerial color infrared (CIR) imagery to detect and monitor weed cover dynamics in this system. To our knowledge, this is the first robust and cost-effective method to distinguish annual weed grasses within an annual grass community. There is much potential application for this approach because it uses (1) a relatively simple multi-temporal classification approach that is not species-specific, (2) aerial imagery as its backbone, which is easily understood by land managers, and (3) a methodology that is easily applied across a large range of remote sensing platforms, including emerging UAS/drone technologies. Moreover, the mapping scale is fine enough to provide early detection of new noxious weed infestations, which is critical for early eradication efforts that can prevent landscape-scale spread of invasives [15, 16].

### Sensitivity of remote sensing mapping approach

We developed this mapping approach in partnership with private ranch managers and conservation professionals to quantify landscape-level effects of management actions on invasion in California grasslands. Previously, we developed means of using remote sensing to monitor field-by-field forage production values in order to support rancher decision-making about grazing regimes [35] and to assess the effectiveness of restoration strategies [32]. This partnership allowed us to select technologies (e.g. aerial imagery) and to develop mapping approaches (multi-temporal, phenologically-based) with land managers in mind.

For the base data, we chose aerial CIR imagery because of its long history of acquisition and use, and because it was already familiar to ranch managers. To allow the most effective comparisons, we standardized the imagery by using the red and infrared bands to calculate values of the Normalized Difference Vegetation Index (NDVI), a classic index of plant greenness [36] that is a good predictor of chlorophyll content in grasslands [57]. Digitized imagery provided a spatial resolution that was fine enough to detect weed patches as small as 1 meter, which is appropriate for the clump size of this vegetation and essential for detecting patch edges [40, 58] and for tracking patch size and shape over time [58, 59]. Even more importantly, aerial imagery could be acquired within precise windows of time at specific phenological transitions when spectral differences between the weed and forage grass groups were greatest. Moderate spatial resolution imagery from satellite platforms such as Landsat may be available for little or no charge, but the grain size (e.g., 30 m) is too coarse to detect the patch dynamics of interest and the schedule for image acquisition is usually too inflexible and infrequent. Finer-scale satellite imagery is now available from numerous commercial companies, but at present is too costly for many ranch managers; future market changes may increase use of such resources by the rangeland management community.

The CIR-derived NDVI imagery well captured the biology of our study site, including the overall reduction in greenness during the 2009 drought. Consistent with our findings in 2004 when we first prototyped this approach [34], weed-dominated patches showed a distinctive signature of directional NDVI change from spring to summer (lower to higher NDVI) that contrasted with forage-dominated patches in which change had the opposite sign (higher to lower NDVI). Thus, the distribution of cover dominated by either or both of two weedy annual grasses—*Elymus caput-medusae* and *Aegilops triuncialis—*was best identified with maximum likelihood supervised multi-temporal classifications that took advantage of the combined NDVI characteristics of the weed group in March (peak spring growth in California’s annual grasslands) and in May (end-of-season senescence).

Most importantly for studies of vegetation change, this mapping approach worked well in both normal and dry years. In semi-arid regions like California, drought is a frequent challenge managers must contend with [60], so weed assessment tools need to be robust to dry conditions. During both normal and low rainfall periods, kappa statistic values describing the accuracy of this classification were good to excellent (0.73–0.92). The mean producer’s accuracy over time (93%) indicated that the maps missed only ~7% of weed-dominated cover. The mean user’s accuracy was a little lower (86.1%), reflecting the primary emphasis on detecting weed cover (i.e., achieving a high producer’s accuracy), but still strong. It indicated that about 14% of the area identified as weed-dominated on the maps might instead be equal parts weeds and forage, or forage-dominated.

Lastly, a key strength of the resulting maps was the new view they provided of weed patch dynamics over large areas at a fine-grained spatial scale. The mapping approach described here distinguished notably more patch detail than previously achieved elsewhere with multi-temporal analysis (e.g., [61, 62]). In use, we found the vegetation classification maps to be so consistent with the landscape that when we loaded maps onto our GPS units and walked through the grasslands monitoring our positions in real-time, the fine-grained map details nearly always matched our field observations. As a tool, these maps offer means of quantifying the persistence of, or fine-grained change in, vegetation types from year-to-year, which is extremely difficult without the assistance of remote sensing methods. The general approach we outline has potential to be applied to weed mapping efforts in many grassland systems.

### Remote sensing recommendations

For those desiring practical advice about how to implement this mapping approach, we highlight some important considerations:

1. The nature and size of the region to be imaged. Capturing an entire site in one image can circumvent the need to stitch smaller images together in image-processing, although methods for co-registering images continue to improve [63]. However, if the region encompasses a weather gradient, sub-portions may need to be imaged on different dates to maintain phenological uniformity.
2. The spatial resolution of the imagery. Our rule-of-thumb is to use a pixel width that is about one-half of the smallest object width to be detected (or about one-quarter of its area) [64]. At our study site, we were interested in vegetation patchiness at the scale of ca. 1 m, so we started with imagery digitized to give pixels that were about 0.5 m x 0.5 m (0.35–0.45 m on a side). Somewhat counterintuitively, finer pixilation can make detection more difficult because it introduces extra variance from fine-scale phenomena such as shadowing within canopies (see nice discussion in review by He, Rocchini [56]).
3. Effective georeferencing. Spatial misalignment will produce artifacts. Where possible, it is useful to install flat permanent or temporary ground control points. In grasslands, permanent structures may be lacking, and trees, shrubs, and telephone poles do not serve well as ground control points. Temporary ground control points (e.g., square fabric pieces painted with a distinct target pattern) can be purchased at surveyor supply stores and stapled onto the ground.
4. Supervised classifications are easier to interpret than unsupervised ones but require ‘training’ examples of the target vegetation. It is essential to acquire coordinates of representative examples in the field with good geospatial precision and accuracy. If examples of the target vegetation are not available, one could use proxy vegetation with a similar phenology, such as vegetation along a seasonal water course that may senescence later than surrounding cover.
5. Number of images. Multiple images from key phenological periods within the same year are better than one. We used two—one at peak spring greenness and one during end-of-season senescence. If only one image can be acquired, our findings suggest that in California grasslands a spring (March) image may be most useful, contrary to what on-the-ground field observations might seem to indicate. Among several reasons for this are that the time window for capturing end-of-season (May) vegetation differences is much shorter and variable in date, and that sudden weather events in May, such as hot dry easterly winds, may scorch vegetation unevenly and introduce additional variability. Images should be acquired at a consistent time of day, preferably close to solar noon, under uniform sky conditions.

### Evaluation of weed cover by management unit

Using the multi-temporal classification described, we were able to (1) produce annual distribution maps of cover dominated by goatgrass and medusahead or by forage at the 1-m scale, (2) evaluate change in cover type over time, and (3) quantify vegetation cover differences among management units. Classifications were conducted at the pixel level in raster format. For field use, the size and shape of patches made of numerous pixels aggregated together can be readily evaluated by eye. Researchers needing more quantitative measures of patch dimensions or their vector outlines could use additional landscape analysis tools (e.g., FRAGSTATS [65]), or object-oriented approaches (e.g., e-Cognition, [66]).

At our study site, the maps revealed clear effects of management on weed distribution, with fence-line contrasts across identical soils highlighting management influence. In management units with little to no grazing, weeds dominated a greater percentage of the area than in grazed units, the proportion of area covered by persistent weeds was 1.6–2.8 times greater, and the proportion of total weed cover that was stable from year-to-year was 1.3–1.4 times larger. In contrast, the area of beneficial forage-dominated cover (%) was greater and more persistent in grazed units. Although livestock may also spread weeds [67], these observations are consistent with indications that sheep-grazing can help control medusahead in some cases [68] and with rancher observations that removal of thick weed thatch by livestock consumption or trampling is important in breaking up weed monocultures. Similarly, Mariotte and colleagues [48] found by manipulating thatch quantities that increased thatch enhances the growth and/or seed production of invasive species in California grasslands, while harming the performance of natives. While our investigation does not delve into the specifics of grazing regime impact on these invasive weeds, our findings demonstrate the promise of this mapping tool in quantifying responses to management actions. In ecological terms, this vegetation analysis highlights the extent to which weed monocultures can form and persist in the absence of disturbance. For weeds such as goatgrass, aggregation in patches can reduce the negative impact of interspecific competition and increase seed output per individual [69].

### Future applications

Fine-scale maps of vegetation cover provide essential data both for land managers and for biologists seeking to understand the complexity of plant communities. It is increasingly recognized that to understand community dynamics it is necessary to assess patterns and controllers across multiple scales [70–73]. The mapping methods developed here for aerial imagery can be extended to digital imagers on unmanned airborne systems (“UAS” or “microdrones”) (reviewed in [74]) to increase the potential for rapid, inexpensive, and user-driven assessment of grassland condition [75, 76]. Ultimately, these remote sensing technologies will enable more data-driven optimization of grassland management, paralleling the application of precision agriculture in crop management [77]. For example, GPS-tracking of animal movement could be combined with fine-scale patch data to gain more insight about how specific grazing regimes and range resources drive vegetation change [78, 79]. Alternatively, such landscape analyses could be used to evaluate broader state-and-transition or gradient models of land cover change [52, 80]. Finally, understanding of vegetation patch dynamics and configuration can illuminate a broad range of spatially-explicit ecological processes (e.g., [81]).

## Supporting information

S1 Fig Comparison of daily precipitation in growing year 2008 (September 1 2007 –August 31 2008) and 2009 (September 1 2008 –August 31 2009) at our study location. (TIF)

S2 Fig Comparison of overall accuracy (%) of classification for four types of NDVI-analogue image inputs processed with both unsupervised (U) and supervised (S) classification algorithms. (TIF)

S3 Fig Producer’s and user’s accuracies for weed patches. (TIF)

S4 Fig Producer’s and user’s accuracies for non-weed patches. (TIF)
