# Supplementary figures and images for: Novel fine-scale aerial mapping approach quantifies grassland weed cover dynamics and response to management
Source: PLoS One. 2017 Oct 9;12(10):e0181665. doi: 10.1371/journal.pone.0181665 (PMC5633334; doi:10.1371/journal.pone.0181665)

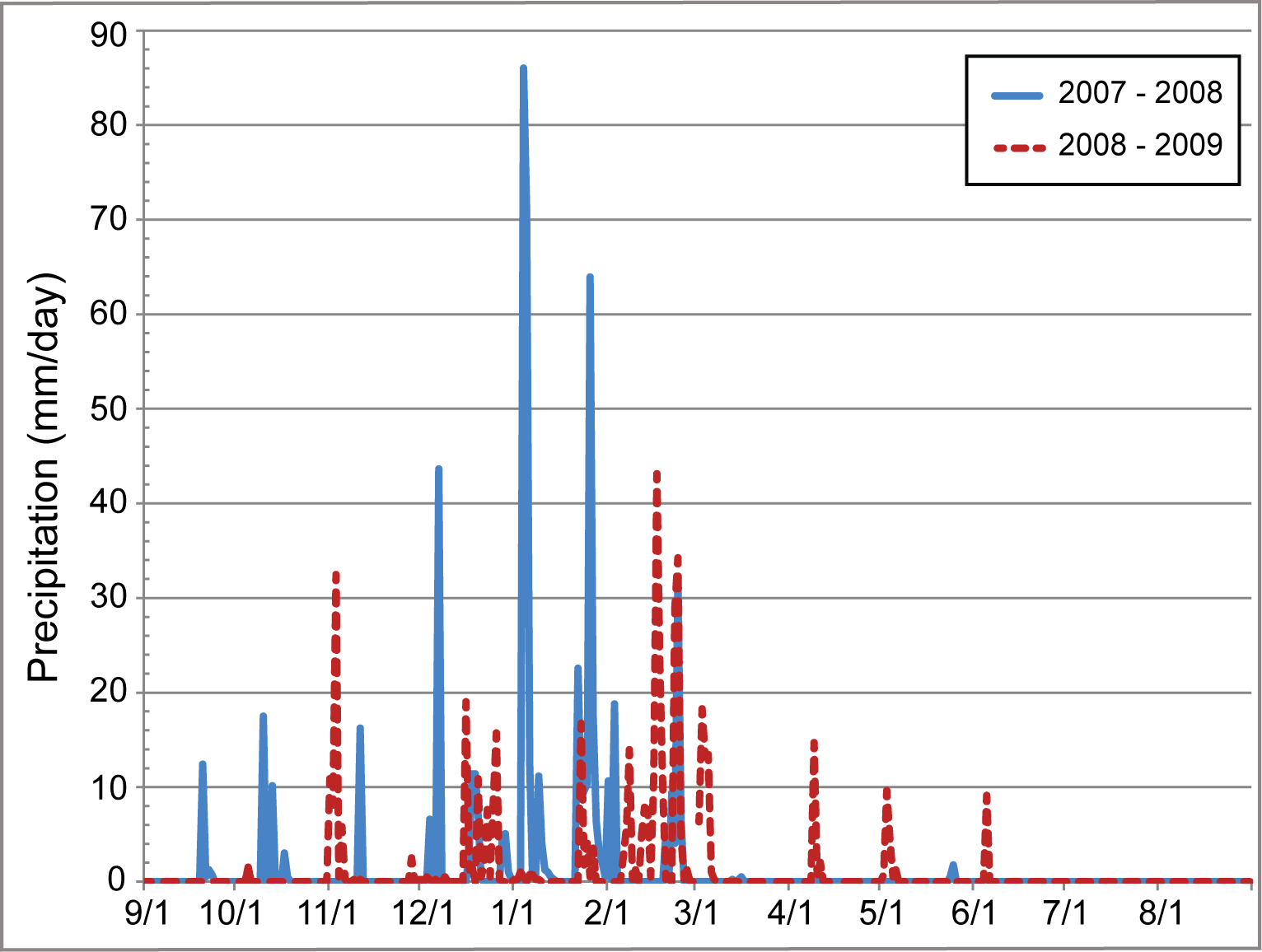

Supplement: S1 Fig — (TIF) [file pone.0181665.s001.tif]

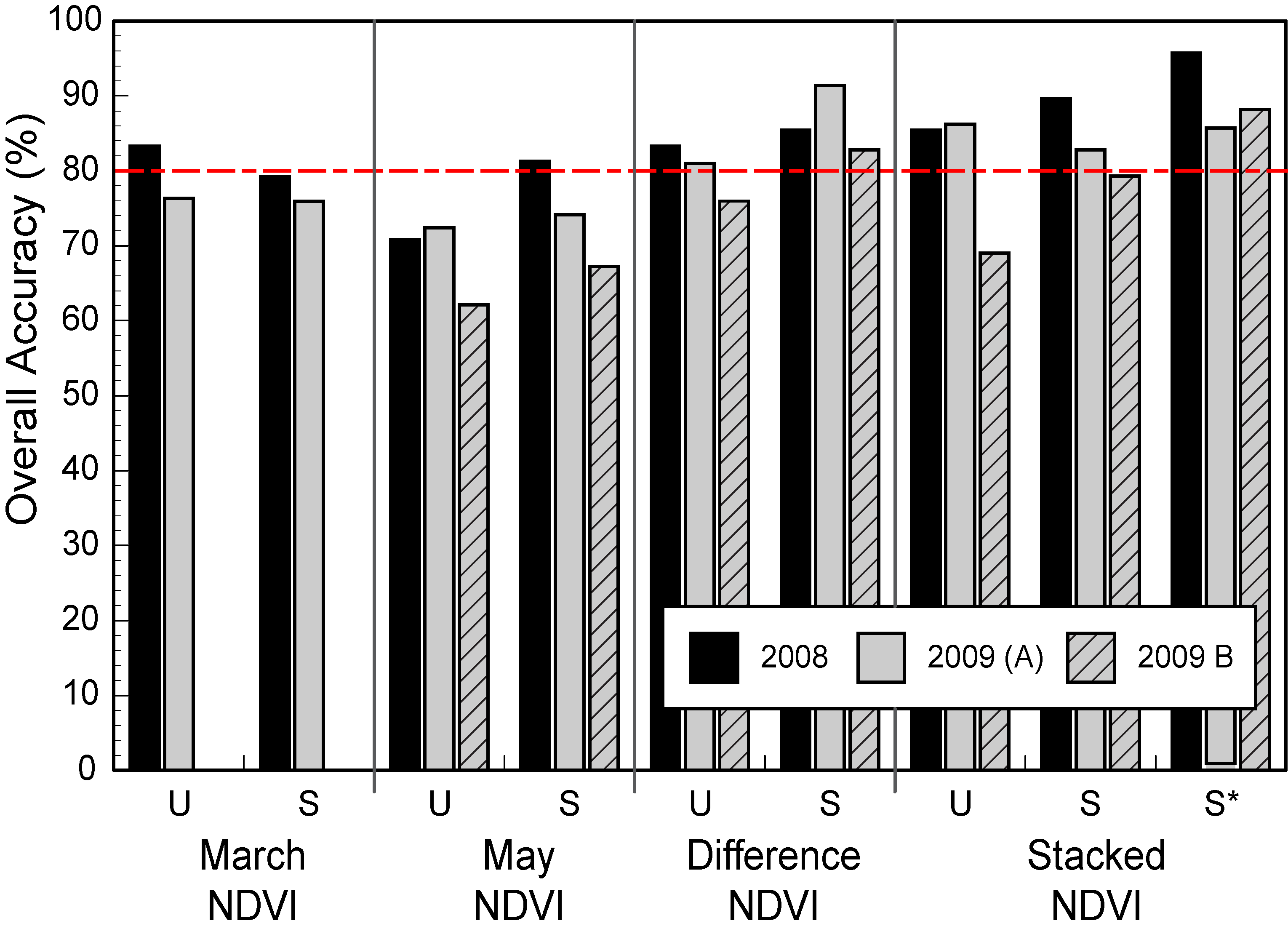

Supplement: S2 Fig — (TIF) [file pone.0181665.s002.tif]

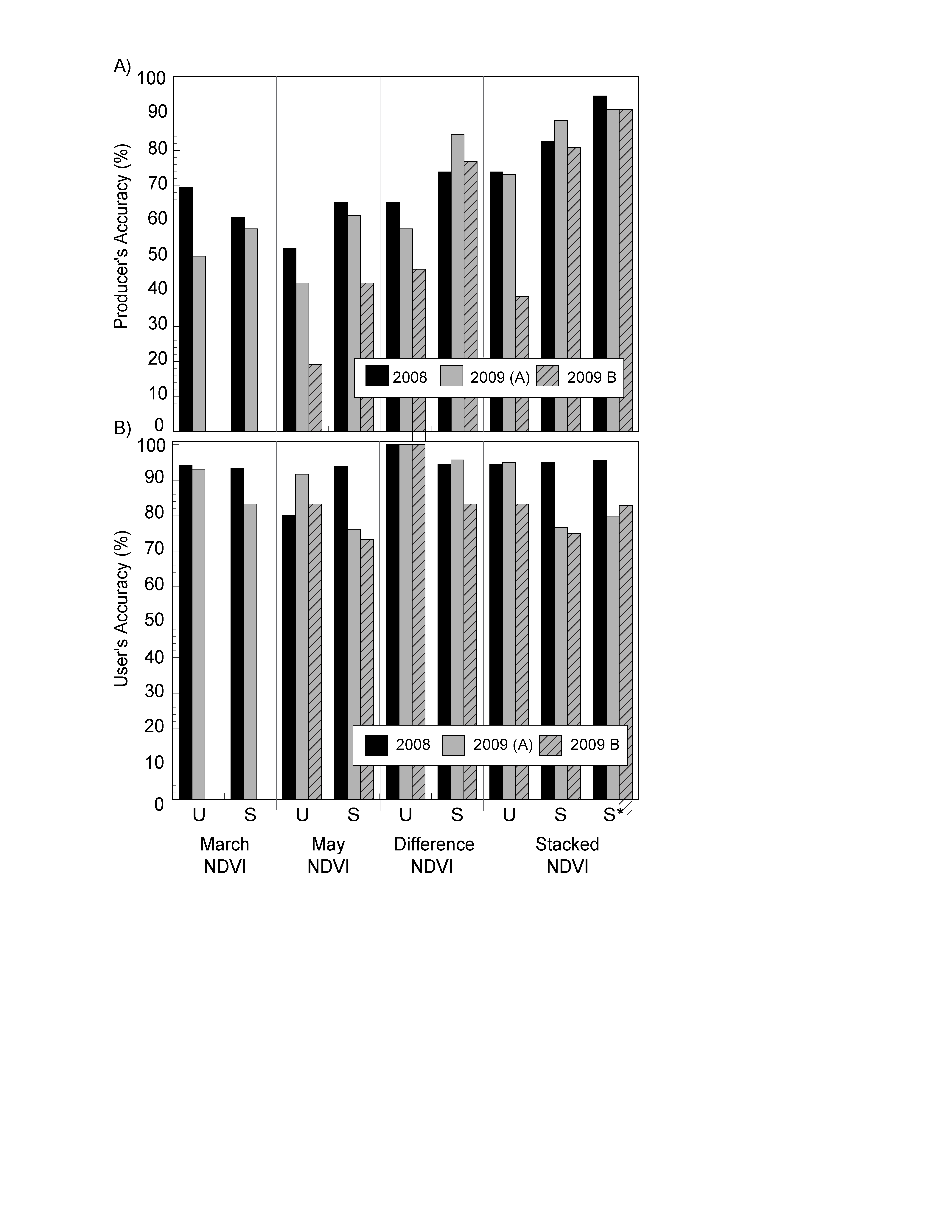

Supplement: S3 Fig — (TIF) [file pone.0181665.s003.tif]

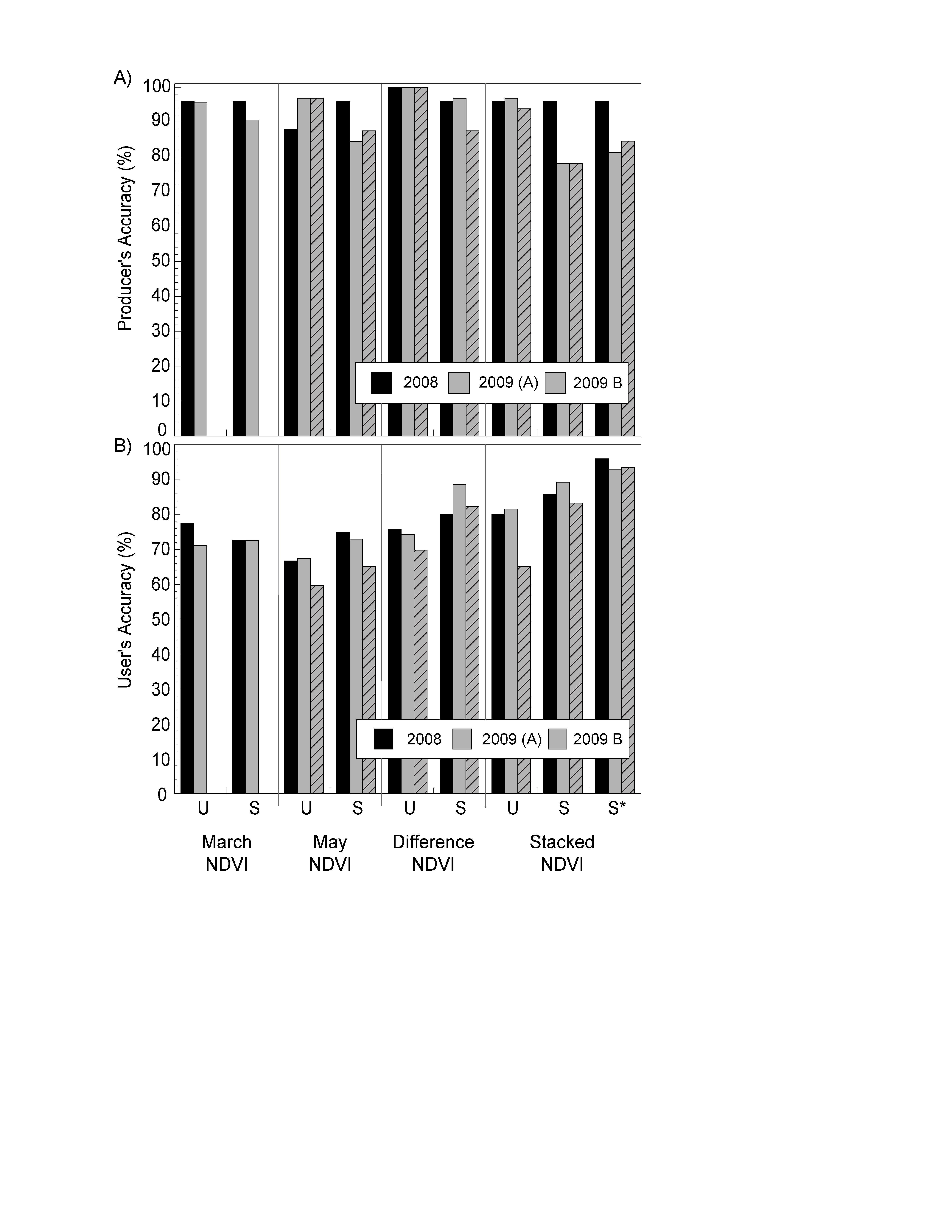

Supplement: S4 Fig — (TIF) [file pone.0181665.s004.tif]
